# The General Medical Council Election

**Published:** 1906-12-15

**Authors:** 


### THE GENERAL MEDICAL COUNCIL ELECTION.

The election of Direct Representatives for England to the General Medical Council has resulted in the return of the following gentlemen to seats at the Council:—Dr. Henry Langley Browne obtained 4,861 votes, Dr. Leonard Strong McManus obtained 4,286 votes, and Dr. Henry Arthur Latimer obtained 4,002 votes.

The unsuccessful candidates were:—Mr. George Jackson, 3,985 votes; Mr. George Brown, 3,773 votes; Mr. Joseph Smith, 2,489 votes; Mr. Frederick John Smith, 2,013 votes; Mr. George Harry Broadbent, 1,785 votes; Dr. Charles Jeremiah Renshaw, 1,088 votes; Dr. John Milson Rhodes, 855 votes.

